# Supplementary figures and images for: Relationship between the Viral Load in Patients with Different COVID-19 Severities and SARS-CoV-2 Variants
Source: Microorganisms. 2024 Feb 20;12(3):428. doi: 10.3390/microorganisms12030428 (PMC10972047; doi:10.3390/microorganisms12030428)

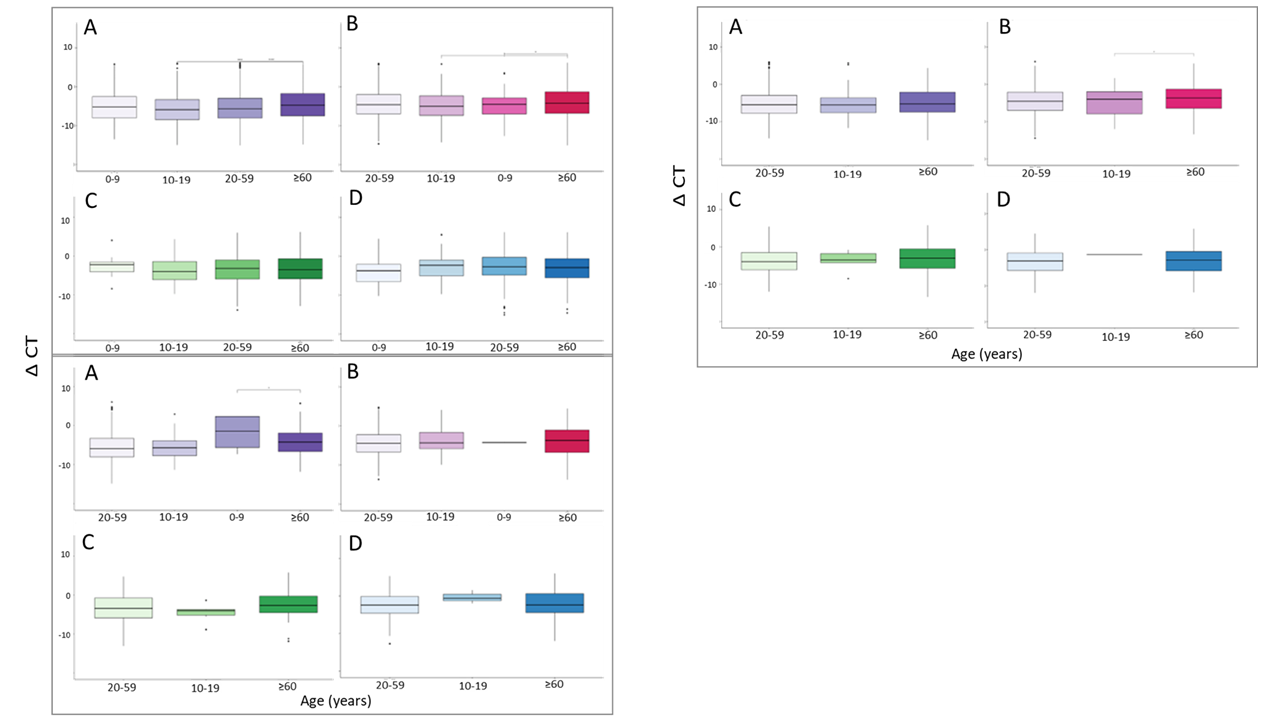

Supplement: Supplementary file 1 [file microorganisms-12-00428-s001.zip › Supplementary figures 05022024/FIGURE S2_PAPER VIRAL LOAD_020224.tif]

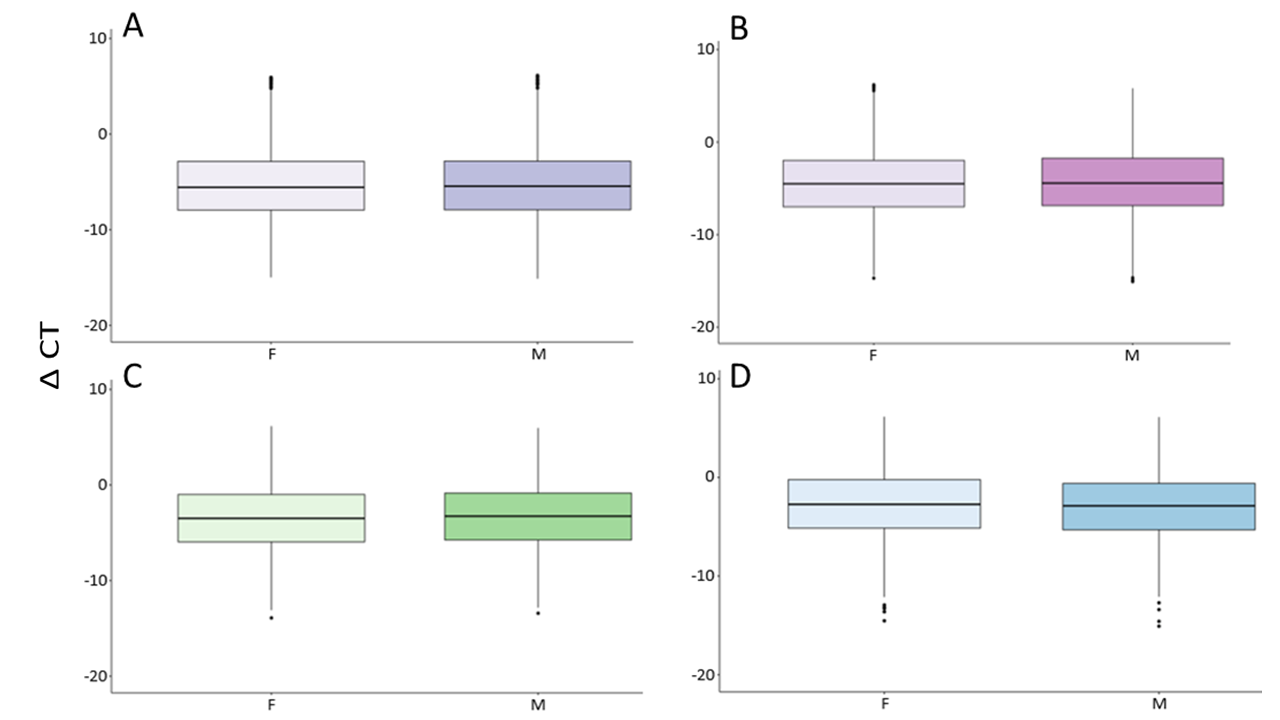

Supplement: Supplementary file 1 [file microorganisms-12-00428-s001.zip › Supplementary figures 05022024/FIGURE S1_PAPER VIRAL LOAD_020224.tif]
